# The expression profile for the tumour suppressor gene *PTEN* and associated polymorphic markers

**DOI:** 10.1054/bjoc.2000.1211

**Published:** 2000-04-27

**Authors:** J A Hamilton, L M D Stewart, L Ajayi, I C Gray, N E Gray, K G Roberts, G J Watson, A V Kaisary, D Snary

**Affiliations:** Applied Development Laboratory, Imperial Cancer Research Fund, St Bartholomew's Hospital, Dominion House, London, EC1A 7BE, UK; Department of Urology, Royal Free Hospital, London, NW3 2OG, UK

**Keywords:** PTEN, microsatellite markers, polymorphisms, prostate, transcription

## Abstract

*PTEN*, a putative tumour suppressor gene associated with prostate and other cancers, is known to be located within the chromosomal region 10q23.3. Transcription of the *PTEN* gives rise to multiple mRNA species. Analyses by Northern blots, using cell lines which express *PTEN* together with cell lines which have lost the *PTEN* or carry a truncated version of the gene, has allowed us to demonstrate that the pseudogene is not transcribed. In addition, 3′ RACE studies confirmed that the multiple mRNA species arising from the gene probably result from the use of alternative polyadenylation sites. No evidence for tissue- or cell-specific patterns of transcription was found. Analysis by 5′ RACE placed the putative site for the start of transcription around 830 bp upstream of the start codon. A map of the location of the *PTEN* gene with a series of overlapping YAC, BAC and PACs has been constructed and the relative position of eight microsatellite markers sited. Two known and one novel marker have been positioned within the gene, the others are in flanking regions. The more accurate location of these markers should help in future studies of the extent of gene loss. Several polymorphisms were also identified, all were within introns. Four of the common polymorphisms appear to be linked. In blood, DNA from 200 individuals, including normal, BPH and prostate cancer patients, confirmed this link. Only two samples of 200 did not carry the linked haplotype, both were patients with advanced prostate cancer. It is possible that such rearrangements within *PTEN* could be evidence of predisposition to prostate cancer in this small number of cases. © 2000 Cancer Research Campaign


*British Journal of Cancer* (2000) **82**(10), 1671–1676
© 2000 Cancer Research Campaign
DOI: 10.1054/ bjoc.2000.1211, available online at http://www.idealibrary.com on IDEAL®

### The expression profile for the tumour suppressor gene *PTEN* and associated polymorphic markers

**JA Hamilton[1,*], LMD Stewart[1,*], L Ajayi[2], IC Gray[1,†], NE Gray[1,‡], KG Roberts[1], GJ Watson[1], AV Kaisary[2] and D Snary[1]**

[1]Applied Development Laboratory, Imperial Cancer Research Fund, Dominion House, St Bartholomew's Hospital, London EC1A 7BE, UK; [2]Department of Urology, Royal Free Hospital, London NW3 2OG, UK

**Summary** *PTEN*, a putative tumour suppressor gene associated with prostate and other cancers, is known to be located within the chromosomal region 10q23.3. Transcription of the *PTEN* gives rise to multiple mRNA species. Analyses by Northern blots, using cell lines which express *PTEN* together with cell lines which have lost the *PTEN* or carry a truncated version of the gene, has allowed us to demonstrate that the pseudogene is not transcribed. In addition, 3′ RACE studies confirmed that the multiple mRNA species arising from the gene probably result from the use of alternative polyadenylation sites. No evidence for tissue- or cell-specific patterns of transcription was found. Analysis by 5′ RACE placed the putative site for the start of transcription around 830 bp upstream of the start codon. A map of the location of the *PTEN* gene with a series of overlapping YAC, BAC and PACs has been constructed and the relative position of eight microsatellite markers sited. Two known and one novel marker have been positioned within the gene, the others are in flanking regions. The more accurate location of these markers should help in future studies of the extent of gene loss. Several polymorphisms were also identified, all were within introns. Four of the common polymorphisms appear to be linked. In blood, DNA from 200 individuals, including normal, BPH and prostate cancer patients, confirmed this link. Only two samples of 200 did not carry the linked haplotype, both were patients with advanced prostate cancer. It is possible that such rearrangements within *PTEN* could be evidence of predisposition to prostate cancer in this small number of cases. © 2000 Cancer Research Campaign

**Keywords**: PTEN; microsatellite markers; polymorphisms; prostate; transcription

Allelic loss at the chromosomal region 10q23–25 has been reported for prostate and several other cancers (Gray et al, 1995; Bose et al, 1998; Cairns et al, 1998; Feilotter et al, 1998; Maier et al, 1998; Robertson et al, 1998). Analysis of loss of heterozygosity (LOH) in microdissected prostate tumours initially placed the minimum region of loss between the markers D10S1644 (AFMa124wd9) and D10S583 (Gray et al, 1998) and was subsequently refined to a region of 400 kb between D10S1765 and D10S541 (Gray, unpublished data). A putative tumour suppressor gene called *PTEN*, *MMAC1* or *TEP1* (Li and Sun, 1997; Li *et al*, 1997; Steck et al, 1997) has been located within this region and was mutated in glioblastomas, endometrial and prostate tumours (Cairns et al, 1997; Rasheed et al, 1997; Tashiro et al, 1997; Feilotter et al, 1998; Gray et al, 1998). In addition, germline *PTEN* mutations have been found to be associated with Cowden's disease (Liaw et al, 1997) and related syndromes such as Bannayan-Zonana syndrome (Marsh et al, 1997).

*PTEN* has regions of homology to chicken tensin and auxilin (Li et al, 1997) and incorporates phosphatase domains which are active against both lipid and protein substrates (Myers et al, 1997; Maehama and Dixon, 1998). Evidence for a tumour suppressor function for *PTEN* comes from experiments in which transfection of *PTEN* into a medulloblastoma cell line null for *PTEN* expression caused inhibition of proliferation and anchorage-dependent growth (Cheney et al, 1998). Total gene disruption resulted in embryonic lethality in mice, whereas animals heterozygous for *PTEN* expression showed hyperplastic-dysplastic changes in epithelial tissues and an increased incidence of tumours (Di Cristofano et al, 1998). The tumour suppressor activity of *PTEN* may result from its demonstrated ability to dephosphorylate the second messenger phosphatidylinositol-3,4,5-triphosphate (PIP3), thereby restricting cell growth and allowing apoptosis to occur (Maehama and Dixon, 1998; Tamura *et al*, 1998). In addition, the ability of *PTEN* to dephosphorylate focal adhesion kinase may prevent cell migration (Tamura *et al*, 1999), and the inactivation of *PTEN* has recently been associated with increased angiogenesis in prostate carcinomas (Giri and Ittmann, 1999).

Analysis of the *PTEN* mRNA species has revealed a complex pattern of different sized transcripts (Steck et al, 1997; Gray *et al*, 1998) some of which could have arisen from alternate splice events. Additionally a pseudogene for *PTEN* (ψ *PTEN*) has been identified on chromosome 9 which has been claimed to be transcribed (Dahia *et al*, 1998). In this paper we investigate origins of the major mRNA species seen on Northern blots and describe BAC/PAC coverage of the region along with the exact location of several polymorphic markers and identification of single nucleotide polymorphisms.

*Received 28 September 1999*
*Revised 17 January 2000*
*Accepted 26 January 2000*

*Correspondence to:* D Snary

†Current address: Smithkline Beecham Pharmaceuticals, Biopharmaceutical Research & Development, New Frontiers Science Park, Harlow, Essex CM19 5AW, UK.
‡Current address: Oxford University Bioinformatics Centre, Institute of Molecular Medicine, John Radcliffe Hospital, Oxford OX3 9DS, UK. *These authors contributed equally to the work.

### MATERIALS AND METHODS

### YAC, BAC and PAC isolation

YAC 821D2 was identified from the CEPH Mega YAC library by using the 'infoclone' program (Chumakov *et al*, 1995) to screen for YACs bearing CA repeat markers spanning the minimal region and a clone obtained from the UK HGMP Resource Centre in Cambridge. BAC and PAC clones were identified from Human BAC pools and RPCI Human PAC libraries (Research Genetics) by polymerare chain reaction (PCR) screening with CA repeat markers. BAC146B18 was isolated as a clone from which we could amplify the T7 (telomeric) end of BAC46B12 and whose centromeric end sequence could be amplified from BAC122L22. Insert size was estimated by restriction enzyme digests using infrequently cutting restriction endonucleases, followed by separation by pulsed field gel electrophoresis using manufacturer's recomended conditions (BioRad).

### Cell lines

The lymphoblastoid line Bristol 8 (BRI8) has been previously described (Gray *et al*, 1998), DU145, PC3 and A172 were from ATCC, and UMUC3 was provided by Prof. M Knowles (Aveyard *et al*, 1998). A second vial of DU145 was obtained from Dr R Schrock (Introgen Therapeutics Inc.). All lines were grown in Dulbecco's modified Eagles medium (DMEM) supplemented with 10% fetal calf serum.

### Exon, intron and microsatellite marker amplification

Exons were amplified as previously described (Gray *et al*, 1998) using primers based on intronic sequences. Primer sequences for Chemical Cleavage of Mismatches (CCM) were obtained from BAC sequence data obtained within the laboratory, and the 5′ end of each 3′ primer was PIG-tailed to facilitate CCM (Brownstein et al, 1996). CCM cycling conditions were as follows: 10 min at 96°C followed by 35 cycles of 96°C for 30 s, 49–60°C for 30 s, 72°C for 5 min in a Perkin-Elmer 9600 thermal cycler and using Taq Gold (Perkin-Elmer). Annealing temperature was dependent on primer pair used.

### BAC sequencing

BACs were prepared using a modified Qiagen midi prep method. Two hundred millilitres cultures were grown in selective medium and processed by the Qiagen midi prep protocol, except that 15 ml of P1, P2 and P3 were used in place of the recommended 4 ml. After centrifugation DNA was precipitated from the supernatant with isopropanol, the pellet washed with 70% ethanol and resuspended in 1 ml of $dH_2O$ (heavy water) and 0.25 ml of 10 M $NH_4Ac$. Non-soluble particles were removed by centrifugation and DNA was ethanol precipitated. After centrifugation, the pellet was redissolved in 2 ml of $H_2O$ and 10 ml of QBT buffer. Qiagen protocols were followed thereafter. The DNA was finally resuspended in a total of 120 µl, and 6–8 µl used per sequence reaction. DNA sequence reactions were performed using Big Dye Terminator Cycle Sequencing Ready Reaction Kits (Perkin-Elmer) and analysed on an ABI377.

### Chemical cleavage of mismatches

CCM in heteroduplexes formed between two different alleles was performed using the hydroxylamine method (Rowley *et al*, 1995). The reaction products were analysed on a 6% acrylamide/urea gel with 12-cm plates at 950 volts for 3.5–4 h on an ABI 377 DNA sequencer. The results were analysed using Genescan 2.1.1 software. When cleavage products were detected, PCR products spanning the relevant interval were purified using Qiagen PCR cleanup columns and sequenced using the amplification primers to determine the exact nature of the mismatch.

### 5′- and 3′-rapid amplification of cDNA ends (RACE)

5′- and 3′-RACE reactions were performed using 0.1–0.2 ng of Marathon-Ready prostate cDNA (Clontech) and Advantage-GC cDNA PCR buffer and polymerase mix (Clontech). PCR reactions were carried out in a 9700 thermal cycler (Perkin-Elmer) in a total volume of 50 µl. Cycling parameters for 5′-RACE were either 94°C for 1 min followed by 35 cycles of denaturation for 30 s and extension for 3 min at 68°C or 'touchdown' PCR where a 30 s denaturation step was followed by 5 cycles of 94°C for 10 s and 72°C for 4 min, 5 cycles of 94°C for 10 s and 70°C for 4 min and 25 cycles of 94°C for 10 s and 67°C for 4 min. When touchdown PCR conditions were used for the primary PCR, the nested PCR parameters were denaturation for 30 s followed by 5 cycles of 94°C for 10 s, 62°C for 30 s, 68°C for 2 min, 5 cycles of 94°C for 10 s, 60°C for 30 s, 68°C for 2 min and 25 cycles of 94°C for 10 s, 59°C for 30 s, 68°C for 2 min. The following primers were used in the primary 5′-RACE reactions: GSP1, 5′-GGCAGAAGCTGCTGGTGGCGGGG; GSP2, 5′-GCCGCCGTGTTGGAGGCAGTAGAAGGGG; GSP3, 5′-AACTGAGCGCAGTCGCGTCCCAGCGC and AP1 (Clontech). In the nested 5′-RACE reactions the primers were NGSP1, 5′-GGAAATGGCTCTGGACTTGGCGG; NGSP2,5′-ACCAACTCTCCGGCGTTCCCAGC; NGSP3, 5′-CAGCGCATAAAGAGTCCTGCCAC and AP2 (Clontech). Cycling parameters for 3′-RACE were 94°C for 1 min followed by 6 cycles of 94°C for 10 s and 67°C for 3 min and 30 cycles of 94°C for 10 s and 64°C for 4 min. The following primers were used in the 3′-RACE reactions in combination with the AP1 and AP2 primers from Clontech: 3′GSP2, 5′-CATCCACAGGGTTTTGACAC; 3′NGSP2, 5′-GGTTGTGTAGCTGTGTCATG; 3′GSP3, 5′-CGGGTTAGGGCAATGGAGGGGAATGC; 3′NGSP3, 5′-CGAGGAATTGGCCGCTGTCACTGC. The RACE products were gel purified (Qiagen) and cloned into pGEM-T Easy Vector (Promega) or sequenced directly.

### RNA and Northern analysis

Messenger RNA was extracted from the cell lines UMUC3, BRI8, DU145 and A172 using FastTrack RNA isolation kit (Invitrogen). Five micrograms of mRNA from each cell line were size-fractionated on a 1% formaldehyde agarose gel and transferred to a Hybond $N^+$ membrane (Amersham) using 20 × SSC (sodium–saline citrate) as transfer buffer. A full length *PTEN* cDNA probe was labelled with $^{32}P$-dCTP using Megaprime DNA labelling system (Amersham). A probe fragment was generated for the 3′UTR region by PCR using primers 5′-GACTGAAAGGTTTTCGAGTCC and 5′-GAGGAGCTACAAAGGACTTGG and labelled with $^{32}P$-dCTP as described previously (Gray et al, 1998). RNA hybridization was carried out using ExpressHyb

*© 2000 Cancer Research Campaign*

hybridization solution (Clontech) according to the manufacturer's instructions. Membranes were washed in 2 × SSC and 0.1% sodium dodecyl sulphate (SDS) at room temperature for 50 min with several changes of wash solution. X-ray film (Fuji) was exposed to the Northern blots in the presence of 2 screens for 1–3 days.

### RESULTS

LOH of the chromosomal region around *PTEN* occurs in several cancer types, and to allow a more detailed analysis of this loss a map of overlapping BACs and PACs was constructed and several microsatellite markers positioned on this map along with the exons/introns of *PTEN* (Table 1). The *PTEN* gene covers approximately 100 kb and the markers D10S2491 and D10S1765 which are located 5′ or telomeric to the gene were found to be identical. D10S608 which has previously been used to map *PTEN* LOH is not located within the designated region and is at least 160 kb telomeric of the *PTEN* gene. Markers AFMa086wg9 (intron 2) and D10S2492 (intron 8) are located within *PTEN*. In addition a further CA repeat was identified from sequencing within intron 8 which was heterozygous in 45% of a sample of blood DNAs from a group of 200 individuals when these were analysed by microsatellite methods.

Comparison of genomic and BAC sequencing with database sequences for intron 1 (Genebank sequence – BAC 265N13) showed that there was probably a deletion in the database submission. Genomic sequences we have obtained from around PTEN have been lodged with Genbank (Accession Nos AF143312, AF143313, AF143314, AF143315, AF143316, AF143317 and AF143318).

In addition to microsatellite markers a search for single nucleotide polymorphisms was undertaken. *PTEN* intronic sequence primers were used to PCR amplify 1–1.5 kb fragments of DNA from 20 normal human blood samples. *PTEN* regions amplified included 1.9 kb downstream of exon 3, approximately 6.7 kb upstream of exon 4, all of intron 4, 1.5 kb downstream of exon 5, and 1 kb downstream of exon 6. These fragments, which covered around 12 kb of sequence in total, were analysed by CCM to identify polymorphisms and potential changes were confirmed by sequencing. Polymorphisms and levels of heterozygosity are listed in Table 2; some of these polymorphisms have been described previously but confirmed by CCM and sequencing during the course of our studies.

Four polymorphic markers located within the *PTEN* gene had a similar frequency of heterozygosity of around 40% and were used to analyse the DNA obtained from the blood of 200 individuals (these included 97 prostate cancer patients, 43 patients with benign prostatic hyperplasia and 60 other non-cancer patients). From this analysis it appeared that two distinct haplotypes existed. For the four markers 1, 4, 5 and 8 respectively (Table 2), the linkage was either G,A,+,G or A,G,–,T. Of the 200 DNAs tested, 45.5% were heterozygous at each of the markers, 12% were homozygous for A,G,–,T haplotype and 41.5% were homozygous for G,A,+,G haplotype. Only two out of 200 individuals did not carry the linked haplotype in their blood DNA and both were cancer patients, their genotypes were A,G,–,G/T and A,G/A,+/–,G/T.

To analyse the complex pattern of transcription found for *PTEN* a number of cell lines were studied. Five cell lines were analysed by PCR for the presence or absence of *PTEN* and *ψPTEN*. To distinguish *PTEN* from *ψPTEN*, primers complementary to intron sequences were used to amplify *PTEN* exons (*ψPTEN* is processed and has no introns). In cell lines where all nine *PTEN* exons were present these exons were sequenced. The B lymphoblastoid cell line BRI8 and the prostate line DU145 contained all *PTEN* exons and carried no mutations. DU145 has been described as carrying a mutation at codon 145 (Li et al, 1997); we were unable to find this mutation in two independent sources of DU145. Consistent with published results the glioblastoma cell line A172 contains only exons 1 and 2, and the bladder cell line UMUC3 has lost all PTEN exons (Li et al, 1997; Steck et al, 1997; Aveyard et al, 1998). PC3 cells used for these studies contained only exons 1 and 2. The presence of the *PTEN* pseudogene in these cell lines was determined using PCR primers from the boundary of exons 3 and 4 and from exon 6, which could only generate a product when no intronic sequences were present. *PTEN* pseudogene sequences were found to be present in all cell lines examined. Multiple *PTEN* transcripts (Steck et al, 1997; Gray et al, 1998) were evident in BRI8 and DU145 cells, but no mRNA transcripts were detected in UMUC3 cells which were null for *PTEN* but positive for the *ψPTEN*. A shortened transcript was detected in the mRNA from the A172 cell line which has only exons 1 and 2 (Figure 1).

**Table 1** Microsatellite and *PTEN* content of YACs, BACs and PACs and their alignment in the region of LOH

| | Approx size (kb) | D10S 608 | D10S 215 | D10S 1765 (2491) | Exon 1 | Exon 2 | AFM a086wg9 | Exons 3–8 | D10S 2492 | 8/9 CA | Exon 9 | D10S 541 |
|---|---|---|---|---|---|---|---|---|---|---|---|---|
| Y738B12 | 1330 | ■ | ■ | □ | □ | □ | □ | □ | □ | □ | □ | □ |
| Y821-D2 | 1141 | □ | ■ | ■ | ■ | ■ | ■ | ■ | ■ | ■ | ■ | ■ |
| P72G8 | 165 | □ | ■ | ■ | □ | □ | □ | □ | □ | □ | □ | □ |
| P274D21 | 150 | □ | □ | ■ | ■ | □ | □ | □ | □ | □ | □ | □ |
| B2F20 | 90 | □ | □ | ■ | ■ | ■ | □ | □ | □ | □ | □ | □ |
| B46B12 | 230 | □ | □ | □ | ■ | ■ | ■ | ■ | ■ | ■ | ■ | □ |
| B60C5 | 160 | □ | □ | □ | □ | □ | ■ | ■ | ■ | ■ | ■ | □ |
| B146B18 | 110 | □ | □ | □ | □ | □ | □ | □ | □ | □ | □ | □ |
| B122L22 | 105 | □ | □ | □ | □ | □ | □ | □ | □ | □ | □ | ■ |

Black squares indicate the presence and white squares the absence of the marker or exon. Primers used for the amplification of the exons can be found in the Genome Database (http://www.GDB.org) with the exception of CA8/9 which was amplified using the primer pair 5′-CAGCACTTTGG-GAGGCTAAG/5′- TTTCACTTAAAACGTGCAGGGG. B146B18 overlaps B122L22 and B60C5 but does not contain a marker or exon.

© 2000 Cancer Research Campaign 

**Table 2** Polymorphisms in *PTEN* and their frequency in normal DNA samples.

| No. | Intron | Position | Polymorphism | Heterozygosity (%) |
|---|---|---|---|---|
| 1 | 1 | – 96 exon 2 | A/G | 40 |
| 2 | 3 | + 329 exon 3 | T/C | 15 |
| 3 | 3 | + 3362 exon 3 | A/C | 6 |
| 4 | 3 | – 1606 exon 4 | A/G | 39 |
| 5 | 4 | + 109 exon 4 | ATCTTins/del(+/–) | 36 |
| 6 | 4 | + 403 exon 4 | T/C | 5 |
| 7 | 7 | + 461 exon 6 | G/A | 6 |
| 8 | 8 | + 32 exon 8 | T/G | 37 |

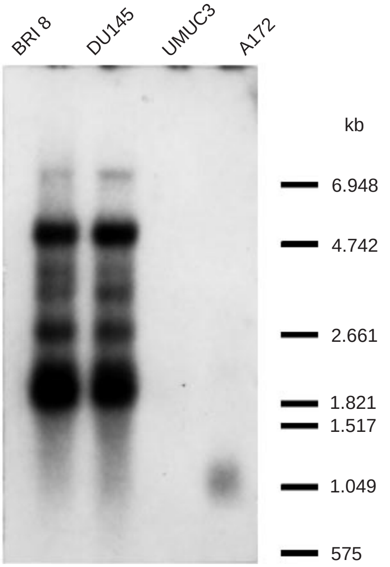

**Figure 1** Northern blot analysis of mRNA from cell lines probed with a *PTEN* cDNA clone. BRI 8 – B lymphoblastoid cell line; DU145 – prostate cell line; UMUC3 – bladder cell line; A172 – glioblastoma cell line

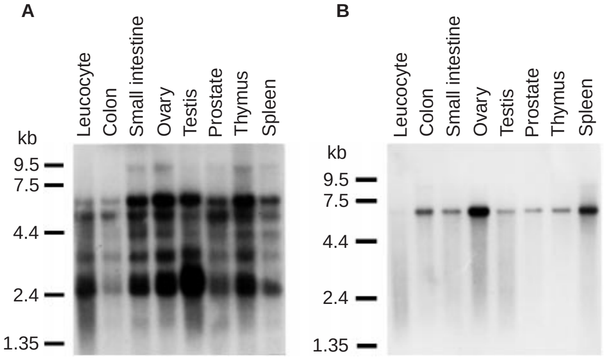

**Figure 2** Northern blot analysis of multiple tissue blots probed with: (**A**) a *PTEN* cDNA; and (**B**) a probe from sequence located 3 kb downstream of the *PTEN* termination codon

Previously we reported that a 121 bp probe from position 338 to 459 upstream of the ATG start codon of *PTEN* hybridized to a single transcript on Northern blots (Gray et al, 1998), which could be evidence of alternate splicing. However, when this probe was used on a Northern blot containing mRNA from BRI8, DU145, UMUC3 and A172 cells a band of approximately 5.5 kb was detected in cell lines that are both positive and negative for *PTEN*. Exact alignment of Northern blots showed that this band was marginally smaller than the expected 5.5 kb transcript detected with a *PTEN* exon 1 probe (data not shown; Gray et al, 1998).

3′-RACE was employed to confirm the use of alternate polyadenylation sites, which would give rise to multiple transcripts, and extension of transcription beyond the end of the published *MMAC1* sequence (GenBank accession U92436). 3′-RACE products generated using primers 3′NGSP2 (located 204 bp downstream of the *PTEN* stop codon) and AP2 terminated with poly-adenosine (poly-A) tails either 290–300 bp downstream from the stop codon and within 20–40 bp of two AAUAAA elements, or where the published *MMAC1* sequence terminates 910 bp from the stop codon (data not shown). 3′-RACE products produced using the primers 3′NGSP3 and AP2 terminated in the vicinity of the end of the *MMAC1* sequence with a poly-A tail. Longer 3′-RACE products were not generated with the conditions used. However, while this work was in progress a search of the GenBank database revealed one EST, IMAGE clone 361374 (Accession Nos AA017563 and AA017584), which extended 271 bp downstream of the end of the *MMAC1* sequence, indicating that transcription can progress beyond the end of the *MMAC1* sequence. Furthermore, a probe from the 3′ untranslated region located 2471–2680 bp downstream of the termination codon, and prior to a group of polyadenylation signals in the genomic sequence, gave a single band on Northern blots equivalent to the largest mRNA species (Figure 2), providing further evidence for transcription extension.

To identify the putative transcription start site and to explore the possibility that long *PTEN* transcripts may result from alternate splicing involving novel 5′ exons, 5′ RACE was performed using three sets of nested primers: GSP1 + NGSP1, GSP2 + NGSP2, GSP3 + NGSP3. The PCR primer set GSP3 + NGSP3, located 860 nt upstream of the longest published cDNA sequence (Li and Sun, 1997), did not result in a PCR product suggesting the coding sequence does not extend into this region. Several PCR fragments were generated using GSP1 + NGSP1 and GSP2 + NGSP2 which displayed sequence identity with previously published sequence (Li and Sun, 1997). The largest fragment extended 827 bp in the 5′ direction from the start codon, 35 bp further than the *TEP1* cDNA sequence. A second fragment also finished at this point while a third finished 824 bp from the start codon.

### DISCUSSION

The *PTEN* gene covers over 100 kb of a 400 kb region of allelic loss common to most prostate tumours (Gray et al, 1995). Although the detection frequency of *PTEN* mutations in prostate

 *© 2000 Cancer Research Campaign*

tumours is lower than anticipated (Cairns et al, 1997; Teng et al, 1997; Feilotter et al, 1998; Gray et al, 1998), the frequency of mutations in other tumour types such as glioblastoma and endometrial carcinoma (Liaw et al, 1997; Rasheed et al, 1997; Tashiro et al, 1997; Teng et al, 1997; Duerr et al, 1998; Risinger et al, 1998), taken together with the data from in vitro cell growth experiments and the targeted disruption of *PTEN* in mice (Di Cristofano et al, 1998), support the view that PTEN is a tumour suppressor gene. We have mapped CA repeat markers D10S608, AFMa086wg9, D10S2491 and D10S2492 precisely around PTEN and identified a number of other common intragenic polymorphisms including a novel CA repeat within intron 8 of *PTEN*. These polymorphisms could prove useful for future studies on *PTEN* and will provide a panel of markers for more detailed mapping of LOH. We have also identified the existence, within the population, of two distinct haplotypes covering this region, although no polymorphisms were found within the coding sequence of *PTEN*.

We have attempted to dissect the complex *PTEN* mRNA transcript profile. Two cell lines, UMUC3 and A172, were used to determine whether a known pseudogene on 9p21 (Dahia et al, 1998) contributes to the pattern of *PTEN* mRNA transcripts. Both cell lines retain the pseudogene but UMUC3 has completely lost both copies of the *PTEN* gene and A172 retains exons 1 and 2 of *PTEN*. On Northern blot analysis no transcript was identified for UMUC3 and an altered message profile was found in A172 cells consistent with the truncated copy of *PTEN* present in this cell line. These results indicate that the pseudogene is not transcribed and does not contribute to any of the transcripts identified in cell lines studied. The pseudogene was originally identified by cloning from RT-PCR implying that transcription was taking place (Kim et al, 1998) and has subsequently been reported as occurring as a major mRNA species (Fujii et al, 1999). Although we cannot exclude the possibility that there could be some cell/tissue-specific transcription of the pseudogene, the similarity of the multiple species seen on Northern blots with mRNA from a variety of different tissues (Gray et al, 1998) and the *PTEN*-positive cell lines described here, taken together with data described by others (Dahia et al, 1998) makes it appear more likely that the pseudogene is not transcribed.

We previously reported that a probe derived from the 5′ sequence of *PTEN* detects only a 5.5 kb transcript whereas full length cDNA probes or probes derived from individual exons all give a complex pattern with major RNA species at 2.1 kb, 2.4 kb and 5.5 kb (Gray et al, 1998). When this probe was used to analyse RNA from *PTEN* null cells a single band was seen in both UMUC3 and A172 which was identical to the pattern seen for *PTEN*-expressing cells. Therefore this 5.5 kb band is distinct from the similarly sized transcript detected with probes derived from the *PTEN* coding region. Consequently it is unlikely to have arisen from alternate *PTEN* splicing and is more likely to be the result of cross-hybridization to an unrelated RNA with some sequence similarity to the *PTEN* 5′ UTR.

Published cDNA sequence of the *PTEN* 5′ UTR extends 790 bp upstream of the ATG translation start site. To determine the region of initiation of transcription a commercial prostate cDNA library was analysed by 5′-RACE. The clones obtained extend the longest previously reported cDNA sequence (Li and Sun, 1997) by 38 bases. Primers derived from genomic sequence 5′ to this region and 5′ to the published *TEP1* cDNA sequence did not produce a PCR product. This suggests that the transcription start site is in the region of the sequence described by Li and Sun (1997), which would give a 5′ untranslated sequence of around 827 bp, with a gene sequence of 1209 bp and the first polyadenylation sites at 60 bp and 290 bp from the translation termination signal. These distances correlate with the size of the smaller mRNA species of 2.1 and 2.4 kb seen on Northern analysis (Gray et al, 1998). The larger 5.5 kb species is likely to result from a further polyadenylation site approximately 3 kb downstream of the second polyadenylation signal. This conclusion is supported by Northern blot analysis where the 5.5 kb mRNA species was identified with a probe from a region 3 kb downstream of the termination codon (Gray et al, 1998). cDNA sequences in the database and 3′ RACE experiments confirm that alternative polyadenylation sites are used and that poly-A tails are added at the equivalent position to the 2.1 and 2.4 kb and that longer species are also transcribed. This analysis taken in conjunction with the analysis of *PTEN* null and *PTEN* truncated cell lines suggests that the complex pattern of mRNAs seen on Northern blots arises largely from alternative polyadenylation sites and that no major contribution to this pattern is made by pseudogene transcription, alternate splicing or cross-hybridization to related gene family members. The significance of the variable 3′ UT sequences is unclear but regulatory elements in this region in other genes have been associated with cell differentiation (Rastinejad and Blau, 1993), mRNA stability particularly in cancer cells (Rajagopalan and Malter, 1997) and tissue-specific expression (Coy et al, 1999).

*PTEN* appears to be an important tumour suppressor gene which is lost in many cancers. In some cases, particularly in glioblastomas and endometrial cancers, the presence of LOH and mutations in the second gene is consistent with the Knudson two-hit hypothesis (Knudson, 1991). In the case of prostate cancer, LOH is common (Gray et al, 1995) but mutations in the remaining gene are rare (Gray et al, 1998), although loss of transcription due to methylation has been reported in some tumour samples (Whang et al, 1998). The detailed genomic map and an analysis of *PTEN* transcripts should assist in the understanding of the genomic changes which contribute to tumour progression by the inactivation of *PTEN* in prostate and other tumours.

### ACKNOWLEDGEMENTS

This work was supported by Zeneca Diagnostics Ltd and Introgen Therapeutics Inc.

### REFERENCES

Aveyard JS, Skilleter A, Habuchi T and Knowles MA (1998) Somatic mutation of *PTEN* in bladder cancer. *Br J Cancer* **80**: 904–908

Bose S, Wang SI, Terry MB, Hibshoosh H and Parsons R (1998) Allelic loss of chromosome 10q23 is associated with tumor progression in breast carcinomas. *Oncogene* **17**: 123–127

Brownstein MJ, Carpten JD and Smith JR (1996) Modulation of non-templated nucleotide addition by *Taq* DNA polymerase: primer modifications that facilitate genotyping. *Biotechniques* **20**: 1004–1010

Cairns P, Evron E, Okami K, Halachmi N, Esteller M, Herman JG, Bose S, Wang SI, Parsons R and Sidransky D (1998) Point mutation and homozygous deletion of *PTEN/MMAC1* in primary bladder cancers. *Oncogene* **16**: 3215–3218

Cairns P, Okami K, Halachmi S, Halachmi N, Esteller M, Herman JG, Isaacs WB, Bova GS and Sidransky D (1997) Frequent inactivation of *PTEN/MMAC1* in primary prostate-cancer. *Cancer Res* **57**: 4997–5000

Cheney IW, Johnson DE, Vaillancourt MT, Avanzini J, Morimoto A, Demers GW, Wills KN, Shabram PW, Bolen JB, Tavtigian SV and Bookstein R (1998) Suppression of tumorigenicity of glioblastoma cells by adenovirus-mediated *MMAC1/PTEN* gene-transfer. *Cancer Res* **58**: 2331–2334

© 2000 Cancer Research Campaign 

Chumakov IM, Rigault P, Le Gall I, Bellanne-Chantelot C, Billault A, Guillou S, Soularue P, Guasconi G, Poullier E, Gros I et al (1995) A YAC contig map of the human genome. *Nature* **377**: 175–297

Coy JF, Sedlacek Z, Bachner D, Delius H and Poustka A (1999) A complex pattern of evolutionary conservation and alternative polyadenylation within the long 3′′-untranslated region of the methyl-CpG-binding protein 2 gene (MeCP2) suggests a regulatory role in gene expression. *Hum Mol Genet* **8**: 1253–1262

Dahia PLM, FitzGerald MG, Zhang X, Marsh DJ, Zheng Z, Pietsch T, von Deimling A, Haluska FG, Haber DA and Eng C (1998) A highly conserved processed *PTEN* pseudogene is located on chromosome band 9p21. *Oncogene* **16**: 2403–2406

Di Cristofano A, Pesce B, Cordoncardo C and Pandolfi PP (1998) *PTEN* is essential for embryonic-development and tumor suppression. *Nat Genet* **19**: 348–355

Duerr EM, Rollbrocker B, Hayashi Y, Peters N, Meyer-Puttlitz B, Louis DN, Schramm J, Wiestler OD, Parsons R, Eng C and von Deimling A (1998) PTEN mutations in gliomas and glioneuronal tumors. *Oncogene* **16**: 2259–2264

Feilotter HE, Nagai MA, Boag AH, Eng C and Mulligan LM (1998) Analysis of *PTEN* and the 10q23 region in primary prostate carcinomas. *Oncogene* **16**: 1743–1748

Fujii GH, Morimoto AM, Berson AE and Bolen JB (1999) Transcriptional analysis of the *PTEN/MMAC1* pseudogene, *ψPTEN*. *Oncogene* **18**: 1765–1769

Giri D and Ittmann M (1999) Inactivation of the *PTEN* tumor suppressor gene is associated with increased angiogenesis in clinically localized prostate carcinoma. *Hum Pathol* **30**: 419–424

Gray IC, Phillips SM, Lee SJ, Neoptolemos JP, Weissenbach J and Spurr NK (1995) Loss of the chromosomal region 10q23–25 in prostate cancer. *Cancer Res* **55**: 4800–4803

Gray IC, Stewart LMD, Phillips SMA, Hamilton JA, Gray NE, Watson GJ, Spurr NK and Snary D (1998) Mutation and expression analysis of the putative prostate tumour-suppressor gene *PTEN*. *Br J Cancer* **78**: 1296–1300

Kim SK, Su LK, Oh Y, Kemp BL, Hong WK and Mao L (1998) Alterations of *PTEN/MMAC1*, a candidate tumor-suppressor gene, and its homolog, *PTH2*, in small cell lung cancer cell lines. *Oncogene* **16**: 89–93

Knudson AG, Jr (1991) Overview: genes that predispose to cancer. *Mutat Res* **247**: 185–190

Li DM and Sun H (1997) *TEP1*, encoded by a candidate tumor suppressor locus, is a novel protein tyrosine phosphatase regulated by transforming growth factor B. *Cancer Res* **57**: 2124–2129

Li J, Yen C, Liaw D, Podsypanina K, Bose S, Wang SI, Puc J, Miliaresis C, Rodgers L, McCombie R, Bigner SH, Giovanella BC, Ittmann M, Tycko B, Hibshoosh H, Wigler MH and Parsons R (1997) *PTEN*, a putative protein-tyrosine-phosphatase gene mutated in human brain, breast, and prostate-cancer. *Science* **275**: 1943–1947

Liaw D, Marsh DJ, Li J, Dahia PLM, Wang SI, Zheng Z, Bose S, Call KM, Tsou HC, Peacocke M, Eng C and Parsons R (1997) Germline mutations of the *PTEN* gene in Cowden disease, an inherited breast and thyroid cancer syndrome. *Nat Genet* **16**: 64–67

Maehama T and Dixon JE (1998) The tumor-suppressor, *PTEN/MMAC1*, dephosphorylates the lipid second messenger, phosphatidylinositol 3,4,5-trisphosphate. *J Biol Chem* **273**: 13375–13378

Maier D, Zhang Z, Taylor E, Hamou MF, Gratzl O, van Meir EG, Scott RJ and Merlo A (1998) Somatic deletion mapping on chromosome 10 and sequence analysis of *PTEN/MMAC1* point to the 10q25–26 region as the primary target in low-grade and high-grade gliomas. *Oncogene* **16**: 3331–3335

Marsh DJ, Dahia PL, Zheng Z, Liaw D, Parsons R, Gorlin RJ and Eng C (1997) Germline mutations in *PTEN* are present in Bannayan-Zonana syndrome [letter]. *Nat Genet* **16**: 333–334

Myers MP, Stolarov JP, Eng C, Li J, Wang SI, Wigler MH, Parsons R and Tonks NK (1997) *PTEN*, the tumor suppressor from human chromosome 10q23, is a dual-specificity phosphatase. *Proc Natl Acad Sci USA* **94**: 9052–9057

Rajagopalan LE and Malter JS (1997) Regulation of eukaryotic messenger RNA turnover. *Prog Nucleic Acid Res Mol Biol* **56**: 257–286

Rasheed BKA, Stenzel TT, McLendon RE, Parsons R, Friedman AH, Friedman HS, Bigner DD and Bigner SH (1997) *PTEN* gene mutations are seen in high-grade but not in low-grade gliomas. *Cancer Res* **57**: 4187–4190

Rastinejad F and Blau HM (1993) Genetic complementation reveals a novel regulatory role for 3′ untranslated regions in growth and differentiation. *Cell* **72**: 903–917

Risinger JI, Hayes K, Maxwell GL, Carney ME, Dodge RK, Barrett JC and Berchuck A (1998) PTEN mutation in endometrial cancers is associated with favorable clinical and pathological characteristics. *Clin Cancer Res* **4**: 3005–3010

Robertson GP, Furnari FB, Miele ME, Glendening MJ, Welch DR, Fountain JW, Lugo TG, Huang HJ and Cavenee WK (1998) In vitro loss of heterozygosity targets the *PTEN/MMAC1* gene in melanoma. *Proc Natl Acad Sci USA* **95**: 9418–9423

Rowley G, Saad S, Giannelli F and Green PM (1995) Ultrarapid mutation detection by multiplex, solid-phase chemical cleavage. *Genomics* **30**: 574–582

Steck PA, Pershouse MA, Jasser SA, Yung WKA, Lin H, Ligon AH, Langford LA, Baumgard ML, Hattier T, Davis T, Frye C, Hu R, Swedlund B, Teng DHF and Tavtigian SV (1997) Identification of a candidate tumour supressor gene, *MMAC1*, at chromosome 10q13.3 that is mutated in multiple advanced cancers. *Nat Genet* **15**: 356–362

Tamura M, Gu J, Danen EH, Takino T, Miyamoto S and Yamada KM (1999) *PTEN* interactions with focal adhesion kinase and suppression of the extracellular matrix-dependent phosphatidylinositol 3-kinase/Akt cell survival pathway. *J Biol Chem* **274**: 20693–20703

Tamura M, Gu J and Yamada KM (1998) Tumor-suppressor *PTEN* inhibition of cell invasion, migration, and growth involvement of focal adhesion kinase. *Mol Biol Cell* **9**: 1429–1429

Tashiro H, Blazes MS, Wu R, Cho KR, Bose S, Wang SI, Li J, Parsons R and Ellenson LH (1997) Mutations in *PTEN* are frequent in endometrial carcinoma but rare in other common gynecological malignancies. *Cancer Res* **57**: 3935–3940

Teng DHF, Hu R, Lin H, Davis T, lliev D, Frye C, Swedlund B, Hansen KL, Vinson VL, Gumpper KL, Ellis L, El-Naggar A, Frazier M, Jasser S, Langford LA, Lee J, Mills GB, Pershouse MA, Pollack RE, Tornos C, Troncoso P, Yung WKA, Fujii G, Berson A, Bookstein R, Bolen JB, Tavtigian SV and Steck PA (1997) *MMAC1/PTEN* mutations in primary tumor specimens and tumor-cell lines. *Cancer Res* **57**: 5221–5225

Whang YE, Wu X, Suzuki H, Reiter RE, Tran C, Vessella RL, Said JW, Isaacs WB and Sawyers CL (1998) Inactivation of the tumor suppressor *PTEN/MMAC1* in advanced human prostate cancer through loss of expression. *Proc Natl Acad Sci USA* **95**: 5246–5250

*© 2000 Cancer Research Campaign*
